# Upregulation of Plasminogen Activator Inhibitor-1 in Irradiated Recipient Arteries and Veins from Free Tissue Transfer Reconstruction in Cancer Patients

**DOI:** 10.1155/2018/4058986

**Published:** 2018-10-04

**Authors:** Bjorn O. Eriksson, Caroline Gahm, Martin Halle

**Affiliations:** ^1^Department of Otorhinolaryngology/Head and Neck Surgery, Karolinska University Hospital, 171 76 Stockholm, Sweden; ^2^CLINTEC, Karolinska Institute, 171 77 Stockholm, Sweden; ^3^Reconstructive Plastic Surgery, Karolinska University Hospital, 171 76 Stockholm, Sweden; ^4^Department of Molecular Medicine and Surgery, Karolinska Institute, 171 77 Stockholm, Sweden

## Abstract

**Background:**

Clinical studies have shown that radiotherapy can induce vascular disease at the site of exposure but is usually not clinically evident until years after treatment. We have studied irradiated human arteries and veins to better understand the underlying biology in search of future treatments. The aim was to investigate whether radiotherapy contributed to a sustained expression of plasminogen activator inhibitor-1 (PAI-1) in human arteries and veins.

**Methods:**

Irradiated arteries and veins were harvested, together with unirradiated control vessels, from patients undergoing free tissue transfer reconstruction at a median time of 90 weeks [5–650] following radiation exposure. Differential gene expression of PAI-1 was analysed, together with immunohistochemistry (IHC) and immunofluorescence (IF).

**Results:**

PAI-1 gene expression was increased in both arteries (*p* = 0.012) and veins (*p* < 0.001) in irradiated compared to unirradiated control vessels. IHC and IF indicated that cells expressing PAI-1 were located in the adventitia of both arteries and veins and colocalized with cells positive for CD68, CD45, and *α*-SMA in arteries and with CD45 and *α*-SMA in veins.

**Conclusion:**

The current study shows a sustained upregulation of PAI-1 in both arteries and veins after exposure to ionizing radiation, indicating a chronic inflammation mainly in the adventitia. We believe that the results contribute to further understanding of radiation-induced vascular disease, where targeting PAI-1 may be a potential treatment.

## 1. Introduction

Radiotherapy, a cornerstone in the treatment of patients with cancer, inevitably involves exposing healthy, surrounding tissue to ionizing radiation. Better overall treatment has led to an increasing number of cancer survivors and consequently more patients suffering from late adverse effects. Recent epidemiological studies have shown that localized cardiovascular disease may occur at the site of exposure, e.g., myocardial infarction or stroke after thorax or neck irradiation, respectively [1–6]. One theory of mechanisms of late vascular morbidity after radiotherapy is that oxidative stress leads to sustained inflammation of blood vessel walls, which is suspected to contribute to the development of atherosclerosis and subsequent thromboembolic events [5, 7, 8]. Previous studies have shown an activation of the nuclear factor kappa-light-chain-enhancer of activated B-cells (NF-*κ*B) inflammatory pathway in irradiated arteries [9] and sustained expression of plasminogen activator inhibitor-1 (PAI-1), a downstream mediator in the NF-*κ*B cascade, in irradiated veins [10]. This may be of relevance for an increased risk of vascular occlusions observed in free-flap reconstructions after radiotherapy [11]. Surgery in previously irradiated patients can furthermore lead to a higher incidence of early and late posttherapeutic complications [12, 13], possibly related to detrimental effects of radiotherapy on the endothelium [14, 15]. PAI-1 has been linked to thrombus formation in the microcirculatory bed [16], which might be a mechanism contributing to general surgical complications as well as flap bed-related complications in the reconstructive setting [10].

The aim of the current study was to corroborate previous evidence of an elevated expression of PAI-1 in previously irradiated arteries and veins by the analysis of the more endothelium-specific Serpine1, instead of Serpine2. By including both arteries and veins in the same analysis, we aimed to gain insight into differences between the respective vessel types and furthermore describe the morphology of PAI-1 expression within the vessel wall, which, to our knowledge, has never previously been described in humans.

## 2. Materials and Methods

### 2.1. Human Tissue Specimens

Seventeen pairs of arterial (group 1) and venous biopsies (group 2) were collected during reconstructive surgery with microvascular free tissue transfer in patients who had received prior radiotherapy due to cancer (see Table 1 for demographics and flap types). For 7 patients, there was enough material for all analyses (arterial and venous, AV), whereas for 5 patients the arteries (A) or the veins (V) were analysed, hence creating two groups of 12 patients (AV + A = group 1 and AV + V = group 2). The irradiated arteries of the head and neck were branches of the external carotid artery, and the veins were branches to the internal jugular vein whereas the donor site arteries and veins were correspondingly sized vessels (e.g., radial artery and vein and peroneal artery and vein). This setup ensured that each individual acted as their own control, and a discerning factor between blood vessels from the two sites was exposure to radiotherapy. The blood vessels were carefully freed from connective tissue under an operating microscope in order not to injure the endothelium. After excision, biopsies for gene expression analysis were placed in RNAlater RNA Stabilization Reagent (Qiagen GmbH, Hilden, Germany), frozen and stored at −80°C until RNA extraction. Biopsies for immunohistochemistry (IHC) and immunofluorescence (IF) were fixed in 10% formalin and embedded in paraffin.

### 2.2. Demography

No major differences were observed in the comparison of background demographic data between the groups (Table 1). There was a male to female preponderance (11 : 6), and the median age was 59 years [30–77]. Median radiation dose was 64 Gy [50–68], and median time since last radiotherapy session was 90 weeks [5–650], with a mean of 150 weeks. Seventeen percent of the patients were current smokers, 12% had a history of cardiovascular disease (peripheral vascular disease, myocardial infarction, cerebral infarction), and the prevalence of hypertension was 24%. All participants signed informed consent sheets. The study was approved by the Ethical Committee of Stockholm and was performed in agreement with institutional guidelines and the principles of the Declaration of Helsinki.

### 2.3. qPCR Analysis

Ribonucleic acid (RNA) extraction was performed using the RNeasy Mini kit (Qiagen GmbH, Hilden, Germany), with an on-column DNase digestion step. RNA quality was analysed via microcapillary electrophoresis via an Agilent Bioanalyzer (Agilent Technologies Inc., Santa Clara, California, USA). The amount of RNA was estimated through UV spectrophotometry with a NanoDrop ND-1000 Uv-Vis Spectrophotometer (NanoDrop Technologies, Wilmington, Delaware, USA). Complementary deoxyribonucleic acid (cDNA) was synthesized from total RNA with Super-Script II reverse transcriptase (Invitrogen Corp., Carlsbad, CA, USA) and stored at −80°C.

### 2.4. Gene Expression Profiling

A TaqMan Endogenous Control Plate was used to check the stability of housekeeping genes. Gene expression results are expressed as delta Ct (dCt) values and delta-delta Ct (ddCt) values. The dCt values were obtained by subtracting the Ct values of the target genes from Ct values of the most stable housekeeping gene, glyceraldehyde 3-phosphate dehydrogenase (GAPDH; veins), and phosphoglycerate kinase 1 (PGK1; arteries). For ddCt values, further subtractions for irradiated vs unirradiated dCt values were performed. The protocol used has previously been described by Halle et al. [8, 10].

### 2.5. Immunohistochemistry

Arterial and venous biopsies were sectioned, deparaffinized, and boiled at 750 W in 0.01 M Na-citrate buffer, then heated at 360 W for another 20 minutes to unmask the epitopes. After blocking of endogenous peroxidase (0.5% hydrogen peroxidase in phosphate-buffered saline (PBS)) and unspecific binding (5% normal goat serum in PBS), each for 30 minutes, primary antibodies (PAI-1; Dako Denmark A/S, Glostrup, Denmark) were applied and samples were incubated at 4°C overnight. After washing, the bound peroxidase was visualized by incubation with a DAB substrate kit (diaminobenzidine, SK-4100, Vector Laboratories Inc., CA, USA). Sections were counterstained with Meyer's hematoxylin, dehydrated, and mounted with DPX (Distrene 80, dibutyl phthalate, xylene; BHD Laboratories Supplies, Poole, UK). PAI-1-positive cells were identified and photographed in light microscopy using a Nikon Eclipse Ni microscope (Nikon Corp, Tokyo, Japan) at 200x–600x power. Image processing was undertaken in Adobe Photoshop (Adobe Systems, San Jose, USA).

### 2.6. Immunofluorescence

After deparaffinization, sections were boiled for 25 minutes in 0.01 M Na-citrate in a 2100 retriever (Electron Microscopy Sciences, Hatfield, PA, USA) to uncloak the epitopes. Serum-free protein block (Dako Denmark A/S, Glostrup, Denmark) was used to decrease nonspecific conjugate binding, and primary antibodies (rabbit polyclonal to PAI-1 (ab66705) 1 : 200, mouse monoclonal to CD45 (ab30470) 1 : 100, mouse monoclonal to CD68 (ab955) 1 : 100, and mouse monoclonal to alpha-smooth muscle actin (*α*-SMA; ab7817) 1 : 100; Abcam, Cambridge, UK) were incubated at 4°C overnight. 2.5% horse serum in PBS was used as a negative control, and for positive controls, normal human kidney (PAI-1) and tonsil (CD68, CD45) were used.

Secondary antibodies (Alexa Fluor 488 goat anti-mouse, and Alexa Fluor 594 goat anti-rabbit, Thermo Fisher Scientific, MA, USA, 1 : 1000) were incubated in the darkness for 30 min. DAPI (4′,6-diamidino-2-phenylindole; Invitrogen Laboratories Inc., CA, USA) (5 mg/ml, 1 : 50000 for 20 minutes) was used to visualize the nuclei. After fixation with Dako Fluorescent Mounting Media (Dako Denmark A/S, Glostrup, Denmark), the sections were stored in a dark environment until IF-microscopy and photography were performed with a laser scanning confocal Nikon Eclipse Ni microscope (Nikon Corp, Tokyo, Japan). Image processing was undertaken in Adobe Photoshop (Adobe Systems, San Jose, USA).

### 2.7. Statistics

Wilcoxon's signed-rank test of paired samples was used to test differences between irradiated and unirradiated vessels for RNA levels. Values of *p* < 0.05 were considered significant.

## 3. Results

### 3.1. Gene Expression

**Figure 1:**
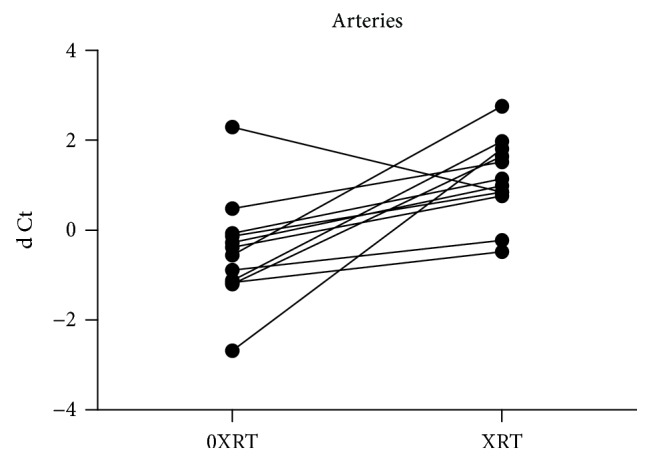
Serpine1 gene (PAI-1) expression is higher in irradiated arteries (XRT) vs unirradiated arteries (0XRT; ddCt), in relation to PGK1 (dCt), *p* = 0.012.

**Figure 2:**
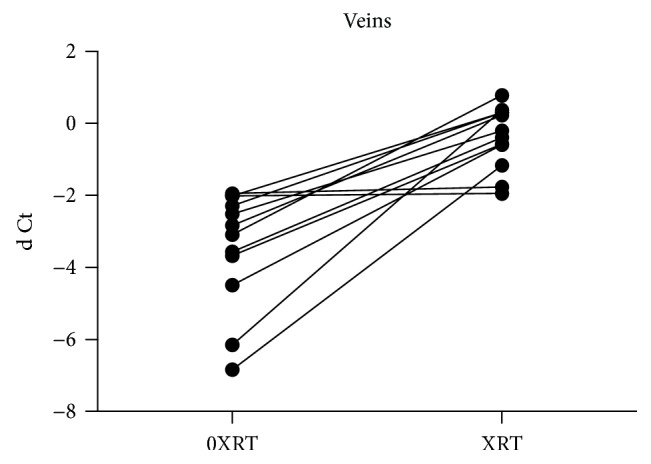
Serpine1 gene (PAI-1) expression is higher in irradiated veins (XRT) vs unirradiated veins (0XRT; ddCt), in relation to GAPDH (dCt), *p* < 0.001.

There was a significant increase of the Serpine1 gene (PAI-1) expression in arteries (*p* = 0.012) and veins (*p* < 0.001) in irradiated compared to unirradiated vessels from the same patient (Figures 1 and 2). The median fold change was 5.05 [0.26–30.0] in arterial samples and 8.32 [1.04–91.3] in the venous samples.

### 3.2. Immunohistochemistry

**Figure 3:**
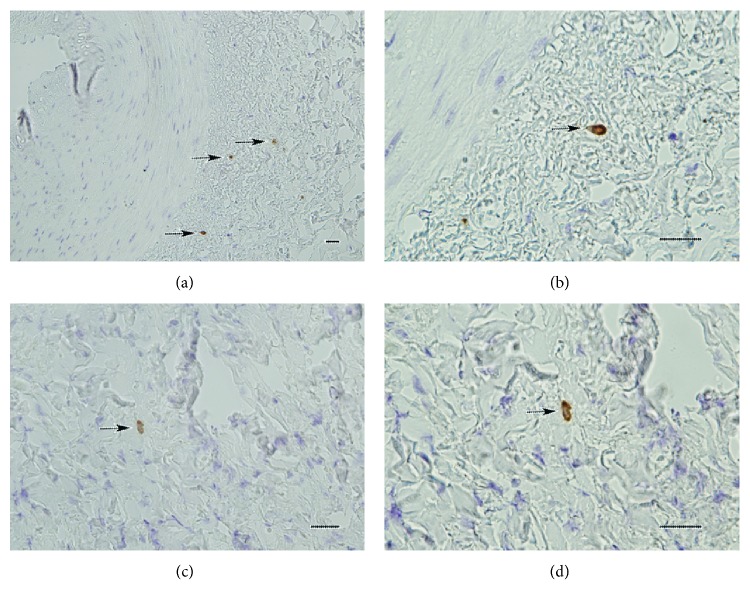
Immunohistochemistry showing PAI-1-positive cells (black arrows) in the adventitia of an irradiated artery (a), with a close-up showing the morphology of one such cell (b). Similarly, PAI-1-positive cells were found in the tunica externa of irradiated veins (c), with (d) a shown close-up of one such cell (scale bar = 50 *μ*m).

Expression of PAI-1 was detected in both irradiated arteries and irradiated veins, mainly in cells located in the adventitia (Figures 3(a)–3(d)). PAI-1-positive cells showed a morphology of mononuclear cells with an oval, or slot-shaped cytoplasm, consistent with the morphology of myofibroblasts and macrophages.

No PAI-1 expression was detected in analysed sections from unirradiated arteries or veins (not shown).

### 3.3. Immunofluorescence

**Figure 4:**
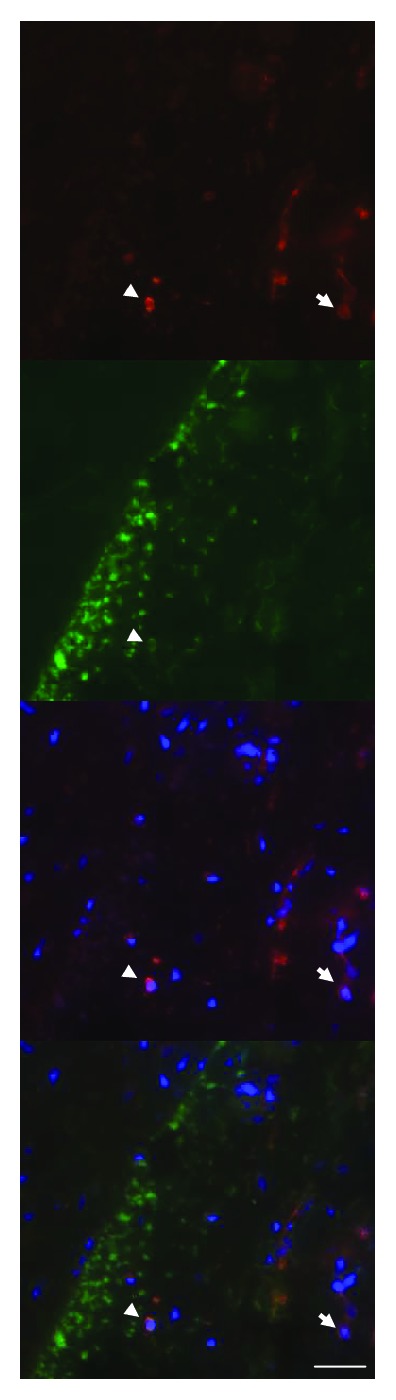
Double-labelling of PAI-1 (red) and CD68 (green) in an irradiated artery. Arrowheads indicate colocalization of PAI-1 and CD68, suggesting that PAI-1 is expressed in macrophages. Arrows indicate cells with PAI-1 expression but no CD68 expression. Nuclear stain (DAPI) is shown in blue (scale bar = 50 *μ*m).

**Figure 5:**
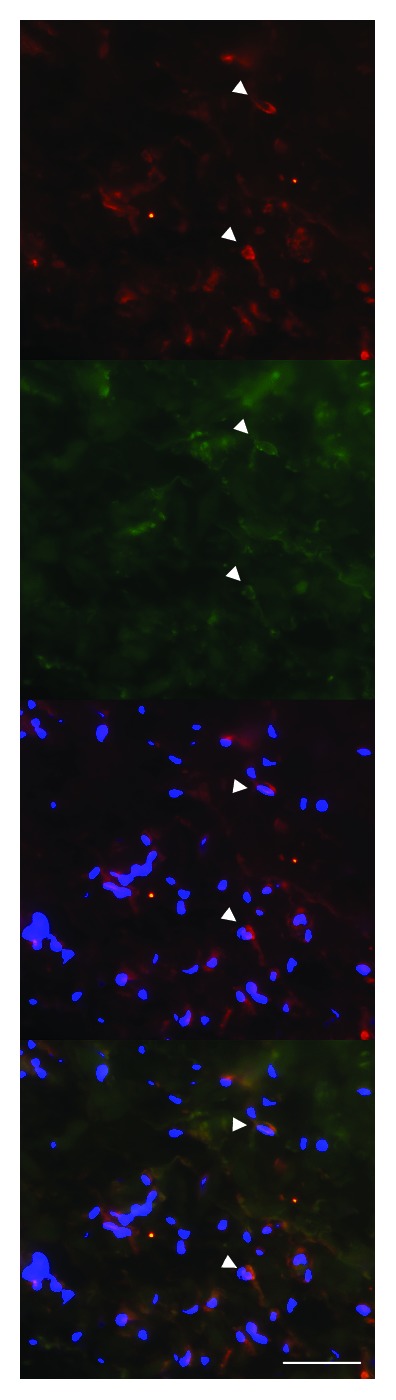
Double-labelling of PAI-1 (red) and CD45 (green) in an irradiated artery. Arrowheads indicate colocalization of PAI-1 and CD45, suggesting expression of PAI-1 in leukocytes. Nuclear stain (DAPI) is shown in blue (scale bar = 50 *μ*m).

**Figure 6:**
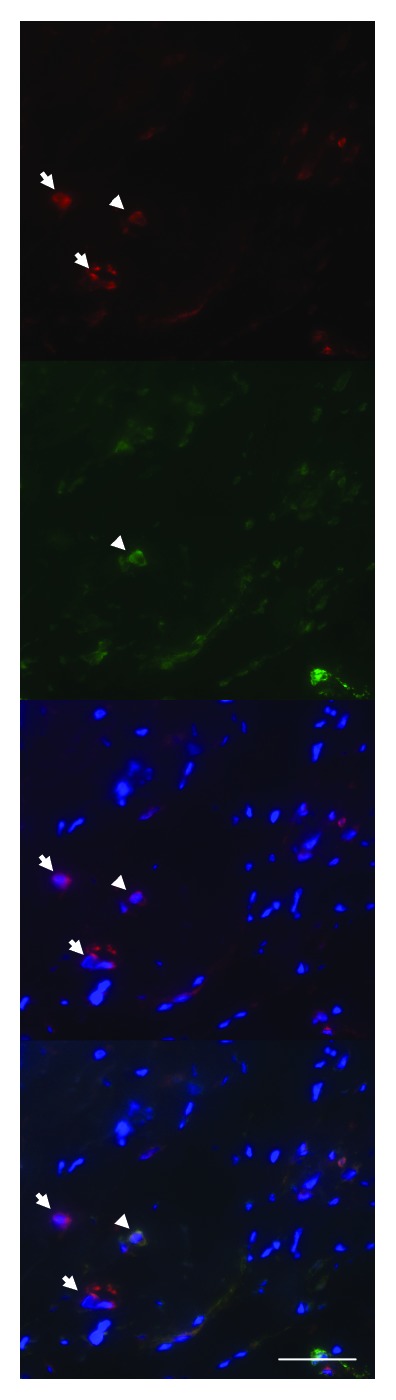
Double-labelling of PAI-1 (red) and *α*-SMA (green) in an irradiated artery. Arrowheads indicate colocalization of PAI-1 and *α*-SMA, suggesting expression of PAI-1 in myofibroblasts. Arrows indicate cells with PAI-1 expression but no *α*-SMA expression. Nuclear stain (DAPI) is shown in blue (scale bar = 50 *μ*m).

**Figure 7:**
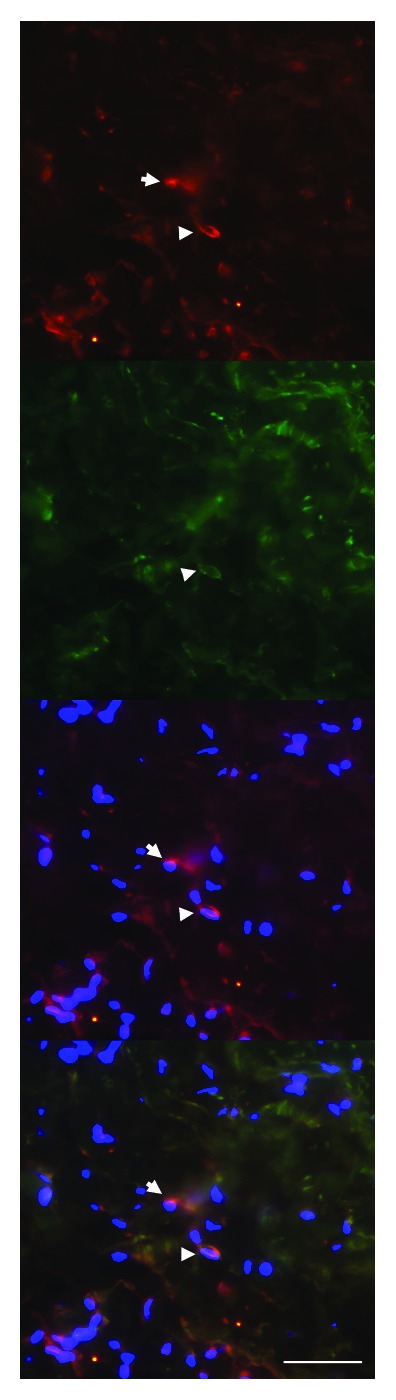
Double-labelling of PAI-1 (red) and CD45 (green) in an irradiated vein. Arrowheads indicate colocalization of PAI-1 and CD45, indicating expression of PAI-1 in leukocytes. Arrows indicate cells with PAI-1 expression but no CD45 expression. Nuclear stain (DAPI) is shown in blue (scale bar = 50 *μ*m).

**Figure 8:**
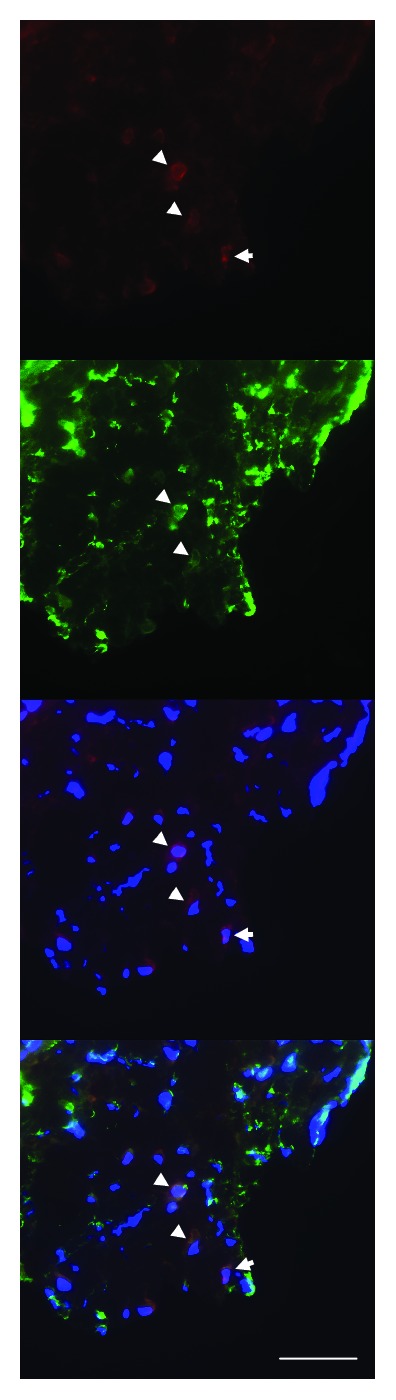
Double-labelling of PAI-1 (red) and *α*-SMA (green) in an irradiated vein. Arrowheads indicate colocalization of PAI-1 and *α*-SMA, indicating expression of PAI-1 in myofibroblasts. Arrows indicate cells with PAI-1 expression but no *α*-SMA expression. Nuclear stain (DAPI) is shown in blue (scale bar = 50 *μ*m).

Colocalization of PAI-1 with CD68, CD45, and *α*-SMA was detected in irradiated arteries. In irradiated veins, colocalization of PAI-1 with CD45 and *α*-SMA was found. Coexpression of PAI-1 and CD68 was not detected in irradiated veins (Figures 4-8). No PAI-1 positivity was found in unirradiated vessels (not shown).

## 4. Discussion

We examined irradiated arteries and veins, compared with unirradiated arteries and veins from the same patient, with gene expression analysis of PAI-1, previously known to be associated with an adverse cardiovascular outcome. Further analysis with IHC and IF confirmed the findings on a protein level and showed that the expression was mainly confined to the adventitia. Previous work has shown upregulation of PAI-1, a downstream mediator in the NF-*κ*B cascade, in irradiated veins after radiotherapy [10]. The increased expression of PAI-1, together with the morphological findings showing its location in the adventitia of both arteries and veins, has never been described before. These results extend previous findings of radiation-induced adventitial inflammation seen in arteries [8].

Several clinical studies have shown that patients previously treated with radiotherapy to the head and neck area or the thoracic region have an increased risk of cardiovascular events, most commonly occurring several years after exposure [1, 5, 6]. This radiation-induced cardiovascular disease may account for up to a third of nonmalignant deaths in groups of cancer survivors, where vascular changes histopathologically resemble those seen in classic atherosclerosis, such as intimal thickening and inflammation [5, 6, 17]. Head and neck cancer patients often have other contributing risk factors for cardiovascular disease (smoking, male gender, and hypertension), and the relative risk for stroke and transient ischemic attack is more than doubled in patients receiving radiotherapy [3]. While Russell et al. found a significant 1.5-fold difference in the intima-media ratio (IMR) in irradiated arteries of the neck compared to unirradiated arteries, the difference was no longer significant when the IMR of the radial artery was analysed as a covariate [5]. In a recent study by our group, we demonstrated that it was the outermost layer of the vessel wall (tunica externa or adventitia) that was chronically inflamed and infiltrated with macrophages in radiation-induced arterial inflammation by means of leukotriene signalling [8].

By primarily affecting the breakdown of fibrin clots through inhibition of tissue plasminogen activator (tPA), a serine protease responsible for the conversion of plasminogen to plasmin, and urokinase-type plasminogen activator, PAI-1 is the main inhibitor of the fibrinolytic system. The majority of circulating PAI-1 is released by activated platelets [18]. A deficiency of PAI-1 leads to moderate bleeding disorders, whereas high levels of PAI-1 can lead to thrombophilia. Epidemiological studies and SNP-analyses for the PAI-1 locus 7q22.1 (SERPINE1) indicate an elevated risk for the onset of coronary heart disease in patients with high PAI-1-levels, independent of other risk factors [18, 19]. Age, smoking, obesity, insulin, and stress can contribute to increased levels of PAI-1, suggesting a link between impaired fibrinolysis and metabolic and cardiac diseases [20–22]. Conversely, exercise has been shown to correlate inversely to inflammation (measured with C-reactive protein) and plasma fibrinogen, as well as PAI-1 levels [23–25]. The cause and effects of increased PAI-1 levels in cardiovascular disease are thus intriguing from many perspectives. Elevated levels of PAI-1 in head and neck squamous cell carcinoma (HNSCC) have also been connected to a shorter disease-free survival, particularly when there are signs of concomitant perineural invasion [26].

Through inhibiting plasmin-mediated matrix metalloproteinases (MMP) activation, PAI-1 also has a role in the regulation of wound healing by disrupting the normal balance between extracellular matrix (ECM) deposition by myofibroblasts and its clearance by MMPs, thus orienting the process in a profibrotic direction [14, 27]. It has also been suggested that the normal downregulation of myofibroblasts seen in wound healing does not take place in fibrotic states due to impeded control of mediators, e.g., transforming growth factor beta 1 (TGF-*β*1) and connective tissue growth factor (CTGF) [28], possibly due to epigenetic changes (such as DNA methylation) that may affect apoptosis [6].

In the acute setting after radiotherapy, activation and dysfunction of the endothelium are believed to create a prothrombotic environment in which adhesion molecules facilitate transmigration of leucocytes to the tissues and an up-regulation of NF-*κ*B and other inflammatory mediators [6, 9–12, 15]. Previous work in our institution has indirectly indicated that pathological changes in the microvascular bed of free tissue transfers (free flaps) may be the culprit for postoperative complications such as fistula formation and infection [13] and has described chronic inflammatory changes in irradiated arteries and veins [4, 7–11]. The results may be of particular interest for microvascular reconstructions, since previous studies indicate that there may be a role for tPA during the rare event of a reexploration for vascular complications [11]. The outcome for the clinical cohort from which biopsies were taken has been reported elsewhere [13]. However, the value of this study lies in the fact that targeting PAI-1 could be a potential treatment for a growing population of cancer survivors with an increased risk of localized cardiovascular disease years after radiotherapy. Many targets for pharmaceutical intervention to prevent fibrosis and cardiovascular morbidity after irradiation have been investigated, for example, blocking the renin-angiotensin-aldosterone system through angiotensin-converting enzyme inhibitors and angiotensin receptor blockers or by using statins [29–34]. Tiplaxtinin (PAI-039, a PAI-1-inhibitor) is an interesting pharmacological agent that has been shown to reduce self-renewal and resistance to radiation in tumor-initiating cells in HNSCC [35].

In the chronic setting, inflammation, tissue hypoxia, oxidative stress, and epigenetic changes have been implicated in the pathophysiology of fibrosis [6, 17, 36]. In an irradiated tissue, immune cells secrete inflammatory and profibrotic cytokines such as IL-6, TGF-*β*1, CTGF/CCN2, and PAI-1, stimulating the activation of myofibroblasts and endothelial to mesenchymal transition (EndoMT), with resulting increases of deposition of collagen, fibronectin, and *α*-SMA in the ECM [14, 27, 36–38]. Irradiation also induces the release of bound, latent TGF-*β*1 (LTGF-*β*1) in the ECM through the actions of reactive oxygen species (ROS), and LTGF-*β*1 can analogously be released via proteases such as plasmin, thrombin, and MMPs [6, 14, 15, 28, 39]. Both TGF-*β*1 and CTGF have been shown to have properties that can sustain a fibrotic response through autocrine positive feedback loops affecting the transcription of profibrotic genes through the Smad and Rho pathways [12, 14, 28, 36, 38, 40]. Inhibition of CTGF and PAI-1, as well as ROS-scavengers, has been shown to decrease fibrosis and remodelling in multiple organ systems [6, 12, 36, 38, 41].

CD68+ macrophages have been demonstrated in the adventitia of the arteries, which demonstrated an expanded vasa vasorum and an increased expression of arachidonate 5-lipoxygenase long after treatment [8], indicating a chronic inflammatory response. It has also been suggested that the normal downregulation of myofibroblasts seen in wound healing does not take place in fibrotic states due to the impeded control of mediators, e.g., TGF-*β*1 and CTGF [28], possibly due to epigenetic changes (such as DNA methylation) that may affect apoptosis [6].

This study has its limitations, as the total number of included patients is low. However, the comparison between irradiated and unirradiated blood vessels from the same patients excludes most of the possible confounding factors, although differences in the morphology of blood vessels can differ depending on anatomical location [16]. Preferably, all arteries and veins would be harvested from the same population, but ethical considerations precluded the use of both arteries and veins in some patients. Some vessels could not be harvested safely in order to obtain a biopsy with sufficient material for both gene expression and immunoassays. However, a majority of the patients (7/12) included contributed to the analyses of both arteries and veins.

## 5. Conclusions

Patients receiving radiotherapy in the setting of head and neck or breast cancer are prone to develop long-term inflammatory and fibrotic changes in the vasculature that likely contribute to an increased incidence of cardiovascular disease in these groups years after radiation exposure. Classic risk factors for the development of atherosclerosis and individual radiosensitivity as well as the administered dose likely contribute to individual risks. Theoretically, a PAI-1-inhibitor could have a therapeutic potential to reduce cardiovascular morbidity for patients receiving radiotherapy, as well as affecting features of prognostic importance for patients with HNSCC.

## Figures and Tables

**Table 1:** Demography of the cohorts. Seven patients participated in both the arterial and the venous groups (AV), 5 only in the arterial group (A), and 5 only in the venous group (V). (XRT = radiotherapy; Gy = gray; RFF = radial forearm flap; FIB = fibular flap; ALT = anterior lateral thigh; and DIEP = deep inferior epigastric perforator). Individual patient characteristics are listed in Supplementary Materials (available here).

Variable	Arterial group	Venous group	Total
Number (*N*)	12 (7 + 5)	12 (7 + 5)	17 (7 + 5 + 5)
Male (AV + A or V)	8 (6 + 2)	9 (6 + 3)	11 (6 + 2 + 3)
Female (AV + A or V)	4 (1 + 3)	3 (1 + 2)	6 (1 + 3 + 2)
Median age (years) [range]	63 [30–77]	59 [30–77]	59 [30–77]
RFF	5	6	7
FIB	6	4	7
ALT	1	1	2
DIEP	0	1	1
Median XRT dose (Gy) [range]	64 [60.3–68]	64 [50–68]	64 [50–68]
Median after XRT (weeks) [range]	118 [5–650]	69 [5–450]	90 [5–650]
Mean after XRT (weeks) [range]	161 [5–650]	117 [5–450]	150 [5–650]
Current smoking (*n*) [%]	2 [17]	2 [17]	3 [18]
Cardiovascular disease (*n*) [%]	1 [8]	2 [17]	2 [12]
Hypertension	4 [33]	3 [25]	4 [24]

## Data Availability

Please contact the corresponding author via e-mail for access to relevant data.

## Supplementary Materials

Supplementary Materials Individual patient characteristics are listed in Appendix 1.
