# Supplementary material for: Upregulation of Plasminogen Activator Inhibitor-1 in Irradiated Recipient Arteries and Veins from Free Tissue Transfer Reconstruction in Cancer Patients
Source: Mediators Inflamm. 2018 Oct 4;2018:4058986. doi: 10.1155/2018/4058986 (PMC6193344; doi:10.1155/2018/4058986)
Supplement: Supplementary Materials — Individual patient characteristics are listed in Appendix 1. [file 4058986.f1.pdf]

Age	Sex	Dose	Time	Smoking	CVD	Flap	A/V/AV	T	N	M	Site	Type
77	F	54	5	Y	Y	RFF	AV	3	0	0	Gingiva	SCC
30	M	68	14	N	N	RFF	AV	3	2b	0	BOT/FOM	SCC
48	M	64	146	N	N	FIB	AV	N/A	N/A	N/A	Eyelid	SC*
50	M	64	90	N	N	RFF	AV	4	2	0	Tongue	MEC
54	M	68	271	N	N	FIB	AV	2	2b	0	BOT	SCC*
64	M	64	215	N	Y	FIB	AV	2	0	0	FOM	SCC
68	M	64	48	N	Y	RFF	AV	4	0	0	Gingiva	SCC
47	F	68	7	N	N	RFF	V	4	0	0	Gingiva	SCC
58	F	50	450	N	N	DIEP	V	N/A	N/A	N/A	Breast	DUC
59	M	68	139	N	Y	FIB	V	3	0	0	BOT	SCC*
59	M	54	7	Y	N	RFF	V	4a	1	0	FOM	SCC
72	M	68	15	N	N	ALT	V	T1§	0	0	Skin	SCC
50	F	60	22	N	Y	RFF	A	3	0	0	Tongue	SCC
62	M	68	290	N	Y	FIB	A	3	0	0	BOT	SCC*
63	M	68	9	N	N	FIB	A	4	2b	0	Gingiva	SCC
63	F	66	650	Y	N	ALT	A	4	1	0	HYP	SCC
73	F	64	170	N	N	FIB	A	2	0	0	Gingiva	SCC*

*Appendix 1 Demography of the patient cohort. Dose = total dose in Gy; Time = weeks elapsed since the conclusion of radiotherapy; Y = yes; N = no; CVD = cardiovascular disease (peripheral vascular disease, myocardial infarction, cerebral infarction); RFF = radial forearm flap; FIB = fibular flap; DIEP = deep inferior epigastric perforator; ALT = anterior lateral thigh; AV = artery and vein; V = vein only; A = artery only; T = tumor extent; N = regional nodes; M = metastasis; SCC = squamous cell carcinoma; BOT = base of tongue; FOM = floor of mouth;N/A = not applicable or known; SC = sebaceous carcinoma; * = flap indication osteoradionecrosis of mandible; MEC = mucoepidermoid carcinoma; DUC = ductal carcinoma; § = initial TNM stage, later presented with regional metastases; HYP = hypopharynx.*
